# Myocardial infarction evaluation from stopping time decision toward interoperable algorithmic states in reinforcement learning

**DOI:** 10.1186/s12911-020-01133-x

**Published:** 2020-06-01

**Authors:** Jong-Rul Park, Sung Phil Chung, Sung Yeon Hwang, Tae Gun Shin, Jong Eun Park

**Affiliations:** 1 College of Information and Communication Engineering, Sungkyunkwan University, Suwon, 16419 Republic of Korea; 2 Department of Emergency Medicine, Yonsei University Gangnam Severance Hospital, Seoul, 06273 Republic of Korea; 3 Department of Emergency Medicine, Samsung Medical Center, Sungkyunkwan University School of Medicine, Seoul, 06351 Republic of Korea

**Keywords:** Electrocardiogram, Myocardial infarction, Least-first-power approximation, Approximate entropy, Stopping time, Reinforcement learning

## Abstract

**Background:**

The Elliot wave principle commonly characterizes the impulsive and corrective wave trends for both financial market trends and electrocardiograms. The impulsive wave trends of electrocardiograms can annotate several wave components of heart-beats including pathological heartbeat waveforms. The stopping time inquires which ordinal element satisfies the assumed mathematical condition within a numerical set. The proposed work constitutes several algorithmic states in reinforcement learning from the stopping time decision, which determines the impulsive wave trends. Each proposed algorithmic state is applicable to any relevant algorithmic state in reinforcement learning with fully numerical explanations. Because commercial electrocardiographs still misinterpret myocardial infarctions from extraordinary electrocardiograms, a novel algorithm needs to be developed to evaluate myocardial infarctions. Moreover, differential diagnosis for right ventricle infarction is required to contraindicate a medication such as nitroglycerin.

**Methods:**

The proposed work implements the stopping time theory to impulsive wave trend distribution. The searching process of the stopping time theory is equivalent to the actions toward algorithmic states in reinforcement learning. The state value from each algorithmic state represents the numerically deterministic annotated results from the impulsive wave trend distribution. The shape of the impulsive waveform is evaluated from the interoperable algorithmic states via least-first-power approximation and approximate entropy. The annotated electrocardiograms from the impulsive wave trend distribution utilize a structure of neural networks to approximate the isoelectric baseline amplitude value of the electrocardiograms, and detect the conditions of myocardial infarction. The annotated results from the impulsive wave trend distribution consist of another reinforcement learning environment for the evaluation of impulsive waveform direction.

**Results:**

The accuracy to discern myocardial infarction was found to be 99.2754% for the data from the comma-separated value format files, and 99.3579% for those containing representative beats. The clinical dataset included 276 electrocardiograms from the comma-separated value files and 623 representative beats.

**Conclusions:**

Our study aims to support clinical interpretation on 12-channel electrocardiograms. The proposed work is suitable for a differential diagnosis under infarction in the right ventricle to avoid contraindicated medication during emergency. An impulsive waveform that is affected by myocardial infarction or the electrical direction of electrocardiography is represented as an inverse waveform.

## Background

The Elliot wave principle assumes the impulsive wave trends including the local maximum value and corrective wave trend below the local maximum value [1]. It was originally considered to predict the financial market trend [2]. Financial market trends and electrocardiograms have impulsive and corrective wave trends that consist of oscillating waves with amplitude deviations. The proposed impulsive wave trends discern the local wave peaks of physiological electric signals from the corrective wave trends.

The performance of a research work in heartbeat detection with the wavelet transform [3] depends on the selection of the wavelet function. The wavelet function requires a threshold definition [3] according to the evaluated number of nearest peaks that could vary among electrocardiograms. Another frequency domain analysis [4] classifies the electrocardiograms into the limited number of classifications from the least-squares support-vector machine [5]. A neural network approach [5] requires the limited number of pre-defined training models under normal heart-beats, and each type of a waveform craves its corresponding model [6]. The proposed impulsive wave trends annotate wave components of heartbeat within a single electrocardiogram, under various types of normal and pathological circumstances without prior knowledge. An annotated electrocardiogram conforms to the standards approved by the HL7 membership in response to the United States Food and Drug Administration [7]. As taking actions in reinforcement learning environments are able to exclude prior knowledge and adaptively learn fluctuating environments with a stochastic policy [8], the actions of the proposed impulsive wave trends constitute numerically tractable reinforcement learning environments. An action then returns a reward value after each action is under a given continual task, or after reaching the final actions within an algorithmic state under a given episodic task [9]. Numerically tractable reinforcement learning processes are constructed with explicit convergence rates in [10]. Reinforcement learning is used as the dominant machine learning in the field of traffic signal control [8] and video game [11].

The proposed work constitutes a novel reinforcement learning environment in medical applications, determined by considering the stopping time decisions with reinforcement learning. The stopping time is defined as the moment of a pre-specified set to be decided on the basis of information [12]. An algorithmic decision utilizing the stopping time optimizes reward as an output among stopping time values [13]. Research works have been conducted by involving stopping time on financial applications [13–16]. Another research work incorporated the stopping time to schedule an advertisement on live social media [17]. The proposed work evaluates the impulsive wave trend for the evaluation of ST-elevation myocardial infarction (STEMI). The stopping time processes need to explain the operating details for improved understanding [13]. The stopping time does not require any knowledge from the future, but it searches independent random variables to define a time sequence that meets a pre-specified condition and determines whether linearly ordered time sequences are defined as stopping time [18].

**Fig. 1:**
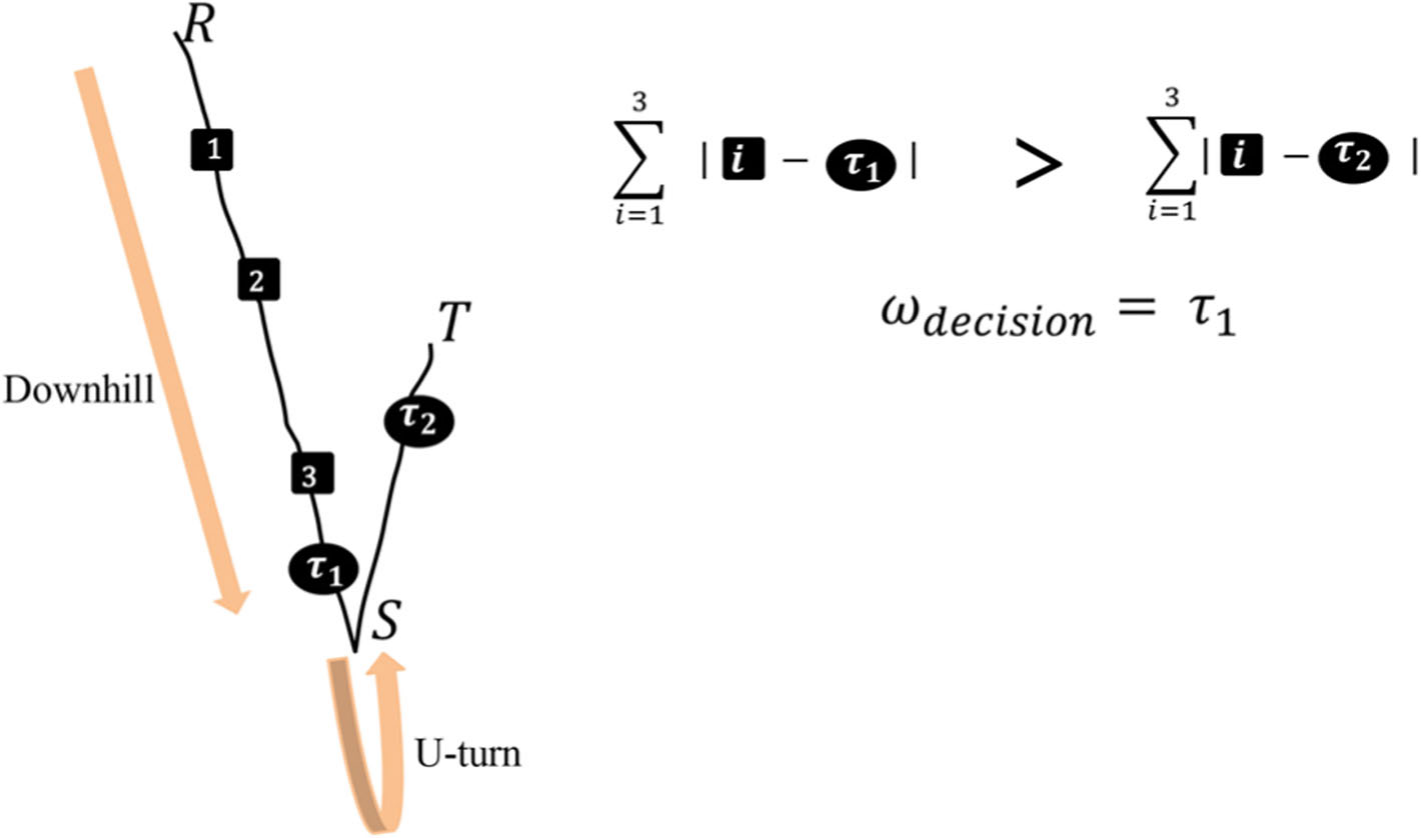
Stopping time decision from downhill U-turn in searching of local minimum value

This research proposes that the decision of stopping time *ω* around the impulsive waves of an electrocardiogram is evaluated from the downhill U-turn process as shown in Fig. 1. When the sampled data in Fig. 1 slides toward the right direction, the trajectory of the data becomes a downhill-shaped slope and U-shaped turn. The local minimum value in Fig. 1, for example, occurs when the output of the downhill U-turn process is maximized, thereby generating the stopping time. In Fig. 1, *i* can be any number in black solid squares, among input data sequence *τ*.

The impulsive wave shape, affected by myocardial infarction (MI) or the location of electrode channel, has an inversed waveform, in comparison to a normal electrocardiogram. The proposed work constitutes another algorithmic state from the approximate entropy to evaluate whether the direction of the impulsive wave is inversed. The state values from both the least-first-power approximation state and approximate entropy state are competitively interoperable by a numerical comparison process to fully explain the current learning processes. Another algorithmic state for the STEMI evaluation result is also interoperable and can determine the shape of the impulsive wave as STEMI affects the impulsive wave. The proposed MI detection technique measures the amplitude between the J point and the approximated baseline point from using a neural network.

Generally, chest pain symptoms are empirically medicated by nitroglycerin (NTG). NTG can be medicated by patients themselves or 911 protocols. However, when the *II*, *III*, and *avF* channels of an electrocardiogram consecutively represent STEMI, there are possibilities of right ventricle (RV) infarction and NTG should not be administrated. Despite the widespread use of electrocardiograms, the prevalence of unrecognized MI is substantial; one in four MIs remains unrecognized [19]. Limitations in the reported results for electrocardiograms and the need for accurate risk assessment in emergency departments (EDs) motivated the development of a novel technique for electrocardiograms.

## Methods

### Experimental setup

MXnet allows the utilization of the central processing unit (CPU) and graphics processing unit (GPU) to ensure more flexibility and acceleration in data processing applications. The processors utilized in the proposed algorithm are 3900X CPU and 2080Ti GPU. The installed version of MXnet is mxnet-cu90mkl. MXnet offers an array manipulating feature, which is suitable for the imperative programming style. The imperative programming style is further accelerated by the automatic parallelization feature of MXnet, similar to the symbolic programming style. The code written in the imperative programming style in MXnet first declares the size of variables; then the variables are identified as the parameters of MXnet libraries or functions to be scheduled for the automatic parallelization feature.

Each electrocardiogram in the experimental dataset consists of 12-channel extensible markup language (XML) waveforms, and lasts for approximately 10 s with 5000 sampled data. The experimental datasets, in XML format, are converted to comma-separated values (CSV) format by the tool from the Cardiovascular Research Grid. An image of a representative beat is converted from XML format electrocardiogram by a basic free version of the XML software tool, called EcgViewer.

The CSV format file is read and imported by the csv and NumPy module in Python. The imported data array is then stored into the memory as NDArray type by MXnet, via a designated processing unit, i.e., CPU or GPU. A normal heartbeat is completed in approximately 1 s. Because the experimental datasets are sampled with 5000 sampled data around 10 s, 300 sampled data are usually enough to represent one QRS complex among several heart beats. The proposed algorithm is set to load only one channel data during an operation for algorithmic flexibility. The experimental datasets include 276 clinical electrocardiograms in CSV format files from 23 patients and 623 clinical electrocardiograms with representative beats from 96 patients.

### Data input

**Fig. 2:**
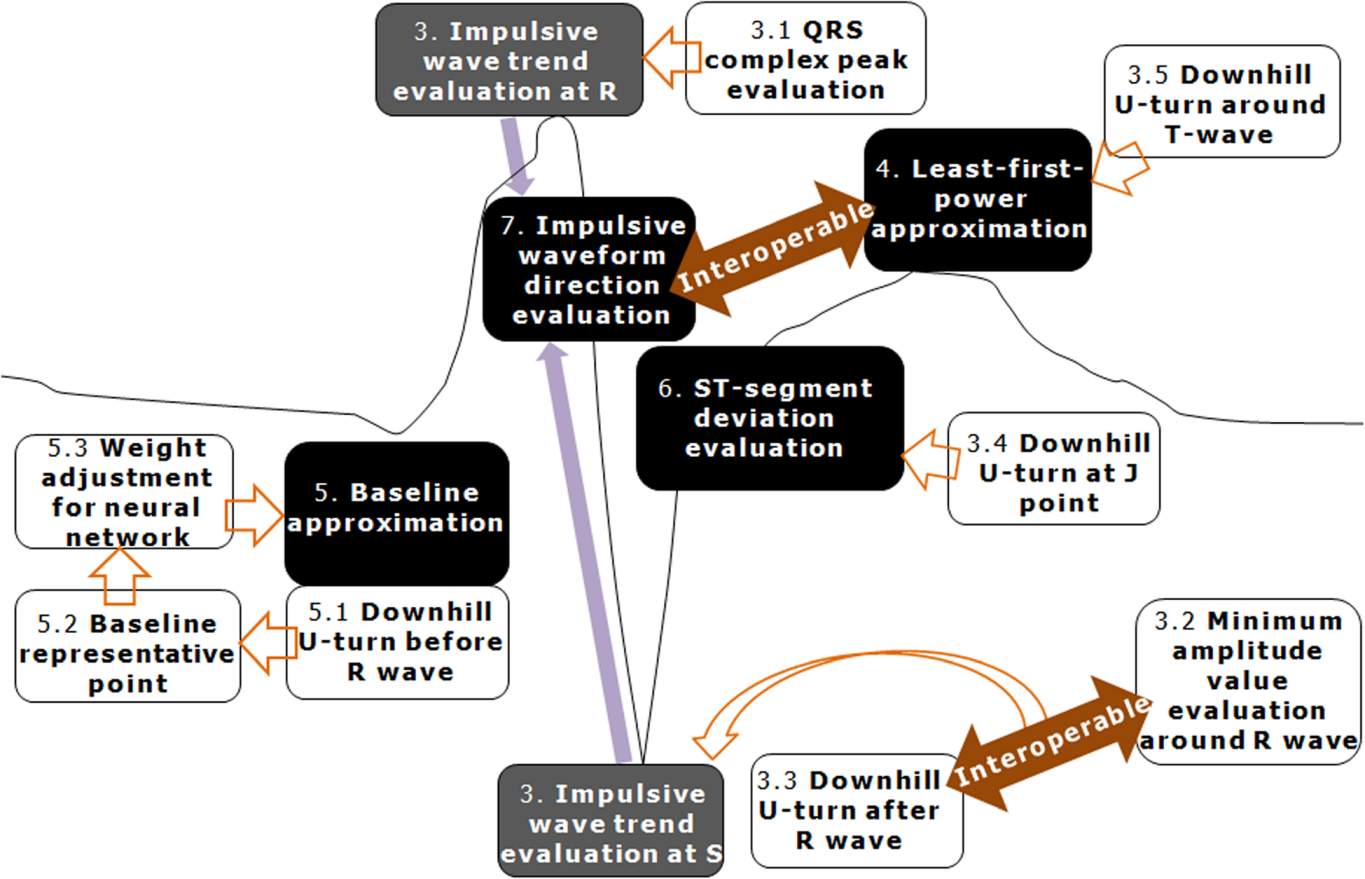
Five proposed evaluation stages in reinforcement learning, marked as dark solid rectangular shapes

The proposed algorithm compensates an oscillation noise component whose amplitude is large enough to hinder the QRS complex waveform. When the amplitude values are same or larger than the value of the absolute average amplitude, they are filtered out. The proposed algorithm iteratively updates a multiplier to be multiplied by the absolute average amplitude. The proposed algorithm stores the electrocardiogram amplitude values below the value, multiplied by the updated multiplier and the absolute average amplitude. The multiplier is iteratively updated until the number of stored amplitude values becomes less than 3000. The data input stage adds the absolute value of the minimum amplitude when the minimum value is less than 0 mV. The data prepared from the data input stage are then ready to process the following proposed stages as shown in Fig. 2.

### Impulsive wave trend evaluation

The impulsive wave trend evaluation stage of the proposed algorithm decides a stopping time point ω for each discovered wave, equivalent to the array indexing numbers. The array indexing numbers of NDArray in MXnet are defined as discrete real numbers. The proposed work addresses an impulsive wave trend distribution as *δ*(*τ*), where *τ* represents the array indexing numbers of an input data sequence.

1 $$ \updelta \left(\uptau \right)=\left\{\begin{array}{c}\ 1,\tau \le \omega\ or\ \tau =\omega\ \\ {}0, otherwise\end{array}\right. $$

The algorithmic decision obtained from the stopping time becomes a deterministic value that represents an actual amplitude value following the input data sequence distribution function. The stopping time finds the deterministic values of the required algorithmic decisions at the impulsive wave trend evaluation stage, and transfers them to the reinforcement learning environment. The input data sequence distribution function *d*(*τ*) represents the intensities of amplitude values for each input data sequence. *δ*(*τ*) and *d*(*τ*) are independent each other and expressed as follows.

2 $$ \omega ={\displaystyle \begin{array}{c} argmax\\ {}\tau \end{array}}\delta \left(\tau \right)d\left(\tau \right) $$

An impulsive wave indicates that *δ*(*τ*)*d*(*τ*) becomes the largest value at the stopping time point *ω*. The stopping time point *ω* exists within the array indexing numbers *τ*, which ranges from zero to the maximum number of array indexes *n*. Each array indexing number is set as *i*.

3 $$ d\left({\bigcup}_0^n{\tau}_i\right)=\delta \left(\omega \right)d\left(\omega \right) $$

#### QRS complex peak evaluation

The proposed algorithm evaluates the amount of slope between the neighboring data only when the slope increases. The maximum slope becomes equivalent to the stopping time. The interval between the neighboring sampled data is set to two data sequences. The input data sequence density function *d*(*i*) represents the amplitude values among array indexing numbers *i*.

4 $$ {\omega}_{QRS}={\displaystyle \begin{array}{c} argmax\\ {}i\end{array}}\left(\frac{d\left(i+ interval\right)-d(i)}{interval}\right) $$

The stopping time *ω*_*QRS*_ is determined when the maximum slope appears between neighbored sampled data at the QRS complex peak evaluation stage. When the array indexing number *τ* matches the stopping time *ω*_*QRS*_, the impulsive wave trend distribution *δ*(*ω*_*QRS*_) becomes 1, as indicated in Eq. (1). The deterministic value of the stopping time decision is then expressed as *δ*(*ω*_*QRS*_)*d*(*ω*_*QRS*_).

The volume of left ventricle at the ending systolic stage is normally increased by 63% [20]. The P wave represents arterial depolarization to circulate blood to the lung. The T wave represents ventricular repolarization, immediately after ventricular depolarization [21]. The QRS complex is modulated by the sodium ion concentration with the conductivity of the corresponding cells, and the T wave is modulated by the concentrations of the calcium and potassium ions with the conductivity of the corresponding cells [22].

A heartbeat cycle including arterial depolarization, ventricular depolarization, and ventricular repolarization is assumed to take 1 s. A subset of 300 sampled data with a sampling rate of 500 Hz achieves the algorithmic stability to include one or less peaks of the QRS complex within the predefined length of the data subset.

Each algorithmic state value follows the policy *π* with the action value, which is defined with the reward value, algorithmic state value after getting the reward value and algorithmic state transition probability with its coefficient *γ* [23, 24]. The proposed algorithm represents an action value *Q*(*s*, *a*) and an algorithmic state value *v* ′ (*s*), as expressed below, where *R*_*s*_ represents a reward value generated at the current algorithmic state *s* with the next state *s’* after taking an action, and policy *π*(*a*, *s*). Each reward value evaluates each action value. Such continual exploration consists of probability distribution between actions [25]. The policy needs to determine the optimized action from any taken actions [9]. The policy *π*(*a*, *s*) determines the quantity of the optimized action value.

5 $$ \mathrm{Q}\left(\mathrm{s},\mathrm{a}\right)=\left[{R}_s+\gamma \mathrm{V}\left({s}^{\prime}\right)\right]P\left({\mathrm{s}}^{\prime }|\mathrm{s},\mathrm{a}\right) $$

6 $$ {\mathrm{v}}^{\prime }(s)=\pi \left(a,s\right)\left[Q^{\prime}\left(s,a\right)-\alpha \left\{\log \left(\pi \left(a,s\right)\right)\right\}\right] $$

The actions of each algorithmic state return a reward value *R*_*s*_ with the next state value and transition probability. The optimized action value *Q* ′ (*s*, *a*) is determined by the maximum action value. The optimized state value is modified by the soft Markov decision process by subtracting the logarithm of the policy [26, 27]. The proposed algorithm updates the state value as *v* ′ (*s*) as expressed in Eq. (6).

The maximum slope appears with the stopping time *ω*_*QRS*_. The QRS complex peak evaluation stage searches every algorithmic state whose transition probability *P*(s^′^| s, a) is 1. The coefficient γ is set as follows.

7 $$ \upgamma =\left\{\begin{array}{c}0,\tau ={\omega}_{QRS}\ \\ {}-1, otherwise\end{array}\right. $$

When the array indexing number matches the stopping time, the action value *Q*(*s*, *a*) shows the amplitude value with the maximum slope between neighboring data. Simultaneously, the action value *Q*(*s*, *a*) becomes optimized as *Q* ′ (*s*, *a*). The updated state value *v* ′ (*s*) treats the policy *π*(*a*, *s*) as 1 and the coefficient *α* as 0, and represents the maximum slope at the stopping time *ω*_*QRS*_.

The updated state values then become the reward values for the upcoming algorithmic states. The next state value *v*(*s*′) is then defined as the difference between the reward values and the 600 amplitude values around the sampled data that represent the reward values. The action value *Q*(*s*, *a*) is then evaluated by setting the coefficient *γ* and transition probability to 1 to preserve the numerical values of the calculation between the reward values and the next state values.

The policy takes the softmax function. *K* ranges from 1 to the number of optimized action values. The coefficient *α* ranges from 0.5 to 100.5, with an increment of 1. From the 101 values of coefficient *α*, an appropriate value is determined when the updated state value *v* ′ (*s*) initially exceeds the action value *Q*(*s*, *a*). When the updated state values *v* ′ (*s*) for any values of coefficient α remains below the action value *Q*(*s*, *a*), the coefficient α is determined to be 0 to maximize the updated state value, as shown in Eq. (6).

8 $$ \pi \left(s,a\right)=\frac{e^{\raisebox{1ex}{$Q\prime {\left(s,a\right)}_k$}\!\left/ \!\raisebox{-1ex}{$\alpha $}\right.}}{e^{\raisebox{1ex}{$Q{\left(s,a\right)}_k$}\!\left/ \!\raisebox{-1ex}{$\alpha $}\right.}+{e}^{\raisebox{1ex}{$Q\prime {\left(s,a\right)}_k$}\!\left/ \!\raisebox{-1ex}{$\alpha $}\right.}} $$

The final outcome of the QRS complex peak evaluation stage represents the amplitude value of the QRS complex peak. Outputs from a learning process that are learned from the previously learned data converge similarly for various types of data as the learning process iterates [28, 29]. The proposed algorithm operates the QRS complex peak evaluation stage twice to reduce any possible chance of a localized maximum value.

#### Minimum amplitude value evaluation around R wave

The actions of the minimum amplitude value evaluation stage searches 50 amplitude values before and after the peaks of R wave are searched. The searching range around the R wave is equivalent to the array indexing numbers τ for the stopping time. A data subset comprising 300 sampled data with a sampling rate of 500 Hz was assumed to achieve the algorithmic stability to include one or less peaks of the QRS complex. The stopping time at the minimum amplitude value evaluation stage is expressed as *ω*_*min*. *amp*_; it represents the array indexing number that indexes the minimum amplitude value around the R wave.

9 $$ {\omega}_{\mathit{\min}. amp}={\displaystyle \begin{array}{c} argmin\\ {}\tau \end{array}}\delta \left(\tau \right)d\left(\tau \right) $$

The impulsive wave trend distribution *δ*(*τ*) and input data sequence distribution function *d*(*τ*) consist of their multiplied form *δ*(*τ*)*d*(*τ*) to represent the deterministic value of the stopping time decision. The next state value *v*(*s*′) is set as the difference value between the deterministic value of the stopping time decision *δ*(*ω*_*min*. *amp*._)*d*(*ω*_*min*. *amp*._) and the amplitude values of the QRS complex peaks. The action values remain the same by keeping the coefficient *γ* and the transition probability constant to represent the actual amplitude values.

#### Downhill U-turn point after R wave

The data sequence of the electrocardiogram data that represents the ending moment of ventricular depolarization has the shape of the trajectory of a downhill moving object a U-shaped turn. A normal waveform of the R wave decreases after the peak of the R wave during ventricular depolarization, and then increases again for ventricular repolarization. The proposed downhill U-turn point stage evaluates the ending moment of ventricular depolarization by searching 50 sampled data as expressed below.

10 $$ {\omega}_{hillUturn}={\displaystyle \begin{array}{c} argmax\\ {}i\end{array}}{\sum}_{i=0}^{24}\left[{\sum}_{j=0}^{24}d\left({\omega}_{QRS}+i\right)-d\left({\omega}_{QRS}+i+24+j\right)\right] $$

The deterministic value of the downhill U-turn point is defined at the stopping point *ω*_*hillUturn*_ by multiplying the impulsive wave trend distribution *δ*() and the input data sequence distribution function *d*(). The reward values *R*_*s*_ for the downhill U-turn point evaluation stage are set as the optimized action values at the QRS complex peak evaluation stage. The next state values are defined as *d*(*ω*_*hillUturn*_) − *R*_*s*_ + 24 with the coefficient *γ* and the transition probability as 1. At the stopping time, the optimized action values are generated.

The second algorithmic state takes an action that searches 150 sampled data before and after the temporarily lower point, which represents the lower value between *v* ′ (*s*) at the minimum amplitude (stage 3.2) and *v* ′ (*s*) at the current first state of downhill U-turn point (stage 3.3). The reward *R*_*s*_ is set to be the same as the amplitude of temporarily lower point, and the next state value *v*(*s*′) is set as the currently searching amplitude. The coefficient value *γ* and the transition probability of the action values are set to 1. The third algorithmic state incorporates the state values from QRS complex peak evaluation (stage 3.1) and both the first and second states of the downhill U-turn point (stage 3.3). The action value of the third algorithmic state is then evaluated as expressed below.

11 $$ Q{\left(s,a\right)}_{3 rd}=\left\{\begin{array}{c}\updelta \left({\omega}_{3 rd}\right)\mathrm{d}\left({\omega}_{3 rd}\right)+\frac{y_{QRS. peak}-{y}_{temp. Low}}{x_{QRS. peak}-{x}_{temp. Low}}\\ {}\updelta \left({\omega}_{3 rd}\right)\mathrm{d}\left({\omega}_{3 rd}\right)+\frac{y_{QRS. downhill}-{y}_{temp. Low}}{x_{QRS. downhill}-{x}_{temp. Low}}\end{array}\right. $$

**Fig. 3:**
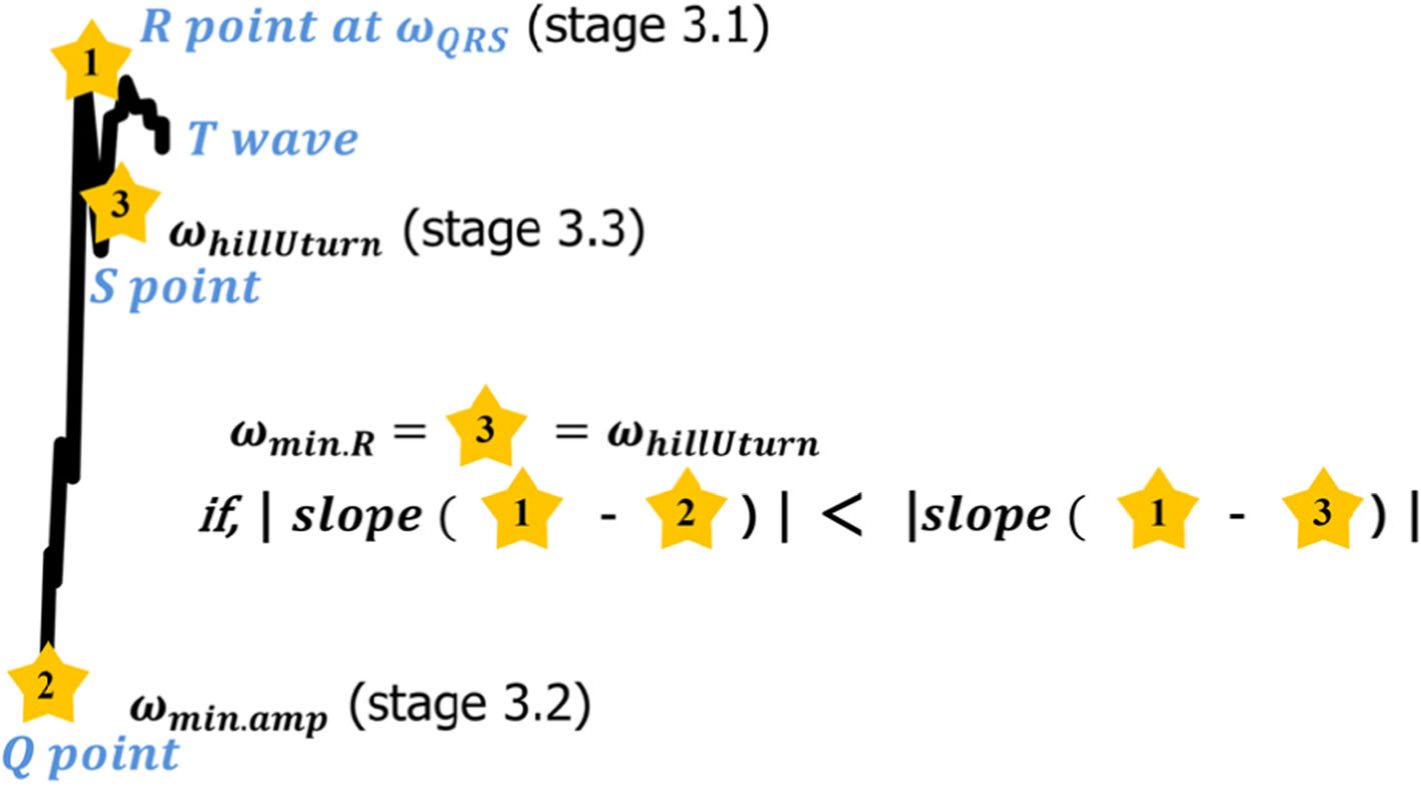
Stopping time decision at downhill U-turn point after R wave

The optimized action value for *Q*(*s*, *a*)_3*rd*_ represents the highest slope, and determines the QRS complex peak. Fig. 3 summarizes how the stages satisfy the S point of the QRS complex appears at stopping time *ω*_*min*. *R*_, equivalent to *ω*_*hillUturn*_.

#### Downhill U-turn at J point

The downhill U-turn at the J point stage searches sampled data within an algorithmic searching range, with 1.5 times the average interval between the QRS complex peak and the minimum amplitude value after the R wave. The J point appears after the R wave, and represents the transition point between the R and T waves of an electrocardiogram. The reward value for the downhill U-turn at the J point stage is set as the minimum amplitude value after the R wave.

The next state value at the downhill U-turn at the J point stage represents the difference between the reward value and the deterministic value of the stopping time decision, expressed in Eq. (12). The algorithmic searching ranges are expressed as *i* and *j*.

$$ {\omega}_{hillUturnJ}= $$

12 $$ {\displaystyle \begin{array}{c} argmax\\ {}i\end{array}}{\sum}_{i=0}^{range}\left[{\sum}_{j=0}^{range}d\left({\omega}_{\mathit{\min}.R}+i\right)-d\left({\omega}_{\mathit{\min}.R}+\mathrm{i}+ range+j\right)\right] $$

The stopping time is added by the half of the algorithmic searching range as the downhill U-turn process considers two algorithmic searching ranges, expressed as *i* and *j*. The ST-segment deviation is evaluated 0.04 s after the J point [30]. Because the time period required for the evaluation of the ST-segment deviation is relatively shorter than the time period of the QRS complex, its range was established between 0.06 and 0.08 s [31]. Because the downhill U-turn at the J point stage adds a half of the algorithmic searching range, the J point defined in the proposed stage locates around the time period range required for the ST-segment deviation evaluation. The stopping time optimizes the action value by adding the next state value. The optimized action value is increased from the reward value. The non-optimized value remains the same as the reward value because the impulsive wave trend distribution *δ*(*ω*_*hillUturnJ*_) is set to 0.

#### Downhill U-turn around T wave

The T wave appears after the J point due to ventricular repolarization, the impulsive wave trend of the T wave is evaluated by the proposed downhill U-turn process. The actions for the downhill U-turn point around the T wave stage includes searching the sampled data with the algorithmic searching range, as expressed below.

13 $$ \mathrm{range}=\mathrm{mean}\left(300+{\omega}_{QRS}-{\omega}_{\mathit{\min}.R}\right)-50 $$

The reward values at the downhill U-turn point around the T wave stage are set as the amplitude values of the J point. The next state values are set as the differences between the reward value and the deterministic value of the stopping time decision. The stopping time index *τ* is the subset of the algorithmic searching range.

14 $$ {\omega}_{Tmax}={\displaystyle \begin{array}{c} argmax\\ {}\tau \end{array}}d\left({\omega}_{hillUturnJ}+\tau \right) $$

The stopping time optimizes the action value, as expressed in Eq. (14). Because the action value needs to represent the actual amplitude value, the coefficient *γ* and the transition probability value are set to 1. The updated state value follows the optimized action value.

The downhill U-turn point around the T wave stage then enters into the next algorithmic state, and searches the sampled data again with its algorithmic searching range, which is 1.5 times the average interval between the QRS complex peak and the minimum amplitude value after the R wave. The algorithmic searching range is limited within the range of 25 and 100 for algorithmic stability to be processed under various electrocardiograms. The downhill U-turn point around the T wave stage follows the downhill U-turn process as introduced in the previous stages. The stopping time is newly defined as below.

$$ {\omega}_{hillUturnT}= $$

15 $$ {\displaystyle \begin{array}{c} argmax\\ {}i\end{array}}{\sum}_{i=0}^{range}\left[{\sum}_{j=0}^{range}d\left({\omega}_{hillUturnJ}+i\right)-d\left({\omega}_{hillUturnJ}+i+ Range+j\right)\right] $$

The stopping time is added a half of the algorithmic searching range as the downhill U-turn process and considers two algorithmic searching ranges, expressed as *i* and *j*. The stopping time defines the second algorithmic state of the downhill U-turn point around the T wave stage. The reward value is set as the amplitude value when *ω*_*hillUturnJ*_ appears. The next state value is set as the difference between the deterministic value of the stopping time decision of the downhill U-turn point around the T wave and the reward value. When the stopping time *ω*_*hillUturnT*_ represents the impulsive wave trend distribution *δ*() as 0, the action value also becomes 0. Because the second algorithmic state, at the downhill U-turn point around the T wave stage, needs to represent the actual amplitude value, the coefficient *γ* and transition probability are set to 1. The updated state value then follows the optimized action value.

### Least-first-power approximation for T wave direction evaluation

The algorithmic searching direction from the J point to the T wave peak is determined by the downhill U-turn process that was evaluated in the second algorithmic state at the downhill U-turn point around the T wave stage. ST depression has a waveform that is shaped as the inversed waveform of the T wave. ST depression occurs from clinical conditions including MI, and represents an inversed shape of the T wave waveform due to ventricular repolarization abnormalities [32]. Because electrocardiography has several channels for measuring the electrical signal data, the channel located at the opposite direction of the main electric current, at the *avR* channel, shows an inversed waveform. The value of the final updated states, at the downhill U-turn point around the T wave stage, represents the direction of the T wave.

16 $$ \left\{\begin{array}{c}{\sum}_i\left|{\omega}_{Tmax}^{first}-{q}_{\omega_i}^{\ast}\right|,{q}_{\omega_i}^{\ast }=d\left({\omega}_{hillUtur nJ}\right)+{\beta}_{hillUtur\mathrm{n}T}\\ {}{\sum}_i\left|{\omega}_{Tmax}^{first}-{r}_{\omega_i}^{\ast}\right|,{r}_{\omega_i}^{\ast }=d\left({\omega}_{hillUtur nJ}\right)-{\beta}_{hillUtur nT}\end{array}\ \right. $$

**Fig. 4:**
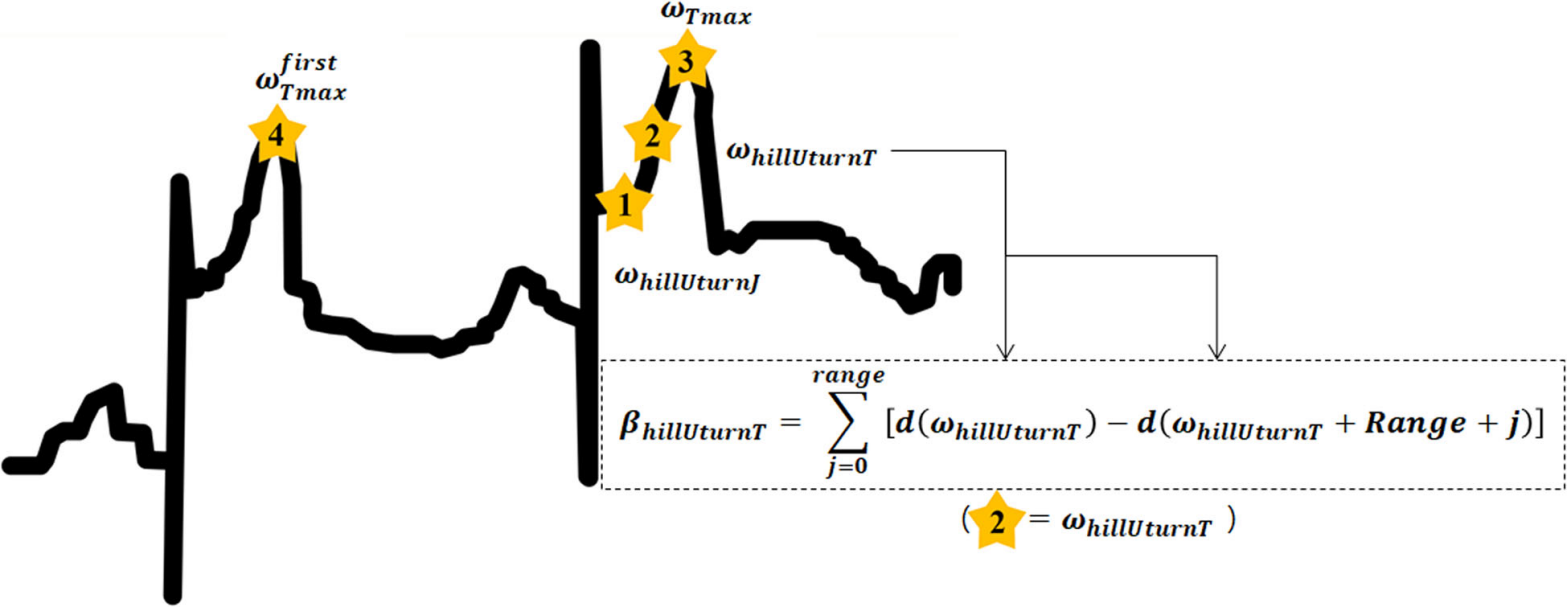
Downhill U-turn process result value at stopping time of downhill U-turn point around T wave

The stopping time *ω*_*hillUturnJ*_ allocates each value with index *i*. The average value between ${q}_{\omega_i}^{\ast }$ and ${r}_{\omega_i}^{\ast }$ has corresponding standard deviation, *β*_*hillUturnT*_, as shown in Fig. 4 and Eq. (16). The average value between ${q}_{\omega_i}^{\ast }$ and ${r}_{\omega_i}^{\ast }$ is expressed as *p*_*ω*_, and it is equivalent to *d*(*ω*_*hillUturnJ*_). When $\left|{\omega}_{Tmax}^{first}-{r}_{\omega_i}^{\ast}\right|$ is equal to or smaller than $\left|{\omega}_{Tmax}^{first}-{p}_{\omega}\right|$, the least-first-power approximation for ${\omega}_{Tmax}^{first}$ out of *p*_*ω*_ becomes ${r}_{\omega_i}^{\ast }$ [33]. When *β*_*hillUturnT*_ has a negative value, the direction from the J point to the T wave peak is upward as a part of a normal T wave waveform, and $\left|{\omega}_{Tmax}^{first}-{q}_{\omega_i}^{\ast}\right|$ tends to become larger than $\left|{\omega}_{Tmax}^{first}-{r}_{\omega_i}^{\ast}\right|$. When *β*_*hillUturnT*_ has a positive value, the direction from the J point to the T wave peak is downward as a part of an inversed T wave waveform, and $\left|{\omega}_{Tmax}^{first}-{q}_{\omega_i}^{\ast}\right|$ tends to become smaller than $\left|{\omega}_{Tmax}^{first}-{r}_{\omega_i}^{\ast}\right|$.

### Baseline approximation

The baseline for the data of an electrocardiogram between the periods of arterial depolarization and ventricular depolarization is utilized as the input vector for the neural network for baseline approximation. The normal atrial depolarization of the heart represents a lower amplitude than that of the QRS complex. The low amplitude values that represent the normal atrial depolarization and ventricular depolarization are vulnerable to noise components. The noise components between atrial depolarization and ventricular depolarization imperil the normal amplitude between them. The noise components impede the prior optimal sampling data intervals [34].

Neural networks generalize and classify input objects into several categories for pattern recognition, signal filtering, or data approximation [1]. Neural networks take data as inputs and process mathematical structures to estimate possible solutions [35]. A neural network adjusts weight values by multiplying the weight and input values to derive the desirable neuron outputs.

#### Downhill U-turn point before R wave

The downhill U-turn point before the R wave stage utilizes the algorithmic searching range at the downhill U-turn point around the T wave stage (stage 3.5), which is 1.5 times the average interval between the QRS complex peak and the minimum amplitude value after the R wave. The reward value is set as the amplitude of the QRS complex peak. The stopping time is evaluated as shown below following the general downhill U-turn process.

$$ {\omega}_{hillUturn-R}= $$

17 $$ {\displaystyle \begin{array}{c} argmax\\ {}i\end{array}}\sum \limits_{i=0}^{range}\left[\sum \limits_{j=0}^{range}d\left({\omega}_{QRS}-i\right)-d\left({\omega}_{QRS}-i- range-j\right)\right] $$

The next state value denotes the difference between the reward value and the deterministic value of stopping time. The coefficient of the next state is − 1. The stopping time optimizes the action value that represents the updated state value of the downhill U-turn point before the R wave stage.

#### Baseline representative point

The baseline representative point offers a part of criteria for MI detection. The downhill U-turn point before the R wave stage utilizes the algorithmic searching range, which is the average interval between the QRS complex peak and the minimum amplitude value after the R wave. The reward value is set as the deterministic value of the stopping time decision at the downhill U-turn point before the R wave stage, equivalent to *δ*()*d*() with the stopping time *ω*_*hillUturn* − *R*_. The stopping time for the baseline representative point stage follows the general downhill U-turn process.

$$ {\omega}_{baselineRP}= $$

18 $$ {\displaystyle \begin{array}{c} argmax\\ {}i\end{array}}{\sum}_{i=0}^{range}\left[{\sum}_{j=0}^{range}d\left({\omega}_{hillUturn-R}-i\right)-d\left({\omega}_{hillUturn-R}-i- range-j\right)\right] $$

The next state value is set as the difference between the reward value and the deterministic value of the stopping time. The coefficient of the next state is − 1; it represents the action value as the amplitude value of an electrocardiogram. The optimized action values from the stopping time become the updated state values.

#### Weight adjustment

Because the baseline before the R wave tends to wander around the neighboring data, the representative value of the baseline needs to be evaluated to detect an impulsive wave trend under the condition of STEMI. The weight adjustment stage utilizes the same algorithmic searching range with the baseline representative point stage. The weight adjustment stage assumes that the baseline interval starts from each baseline representative point, and ends at the location that is left-shifted as much as the algorithmic searching range. The last amplitude value at the baseline interval allocates its weight values, ranging from 0.1 to 2.1. The increment number for the weight values is set to 0.1. The last amplitude value at the baseline interval *d*(*τ*_*last*_) follows the input data sequence distribution function; it is multiplied with the weight value *w*.

19 $$ {baseline}_w=\frac{w\bullet \mathrm{d}\left({\tau}_{last}\right)\ }{0.1\bullet \mathrm{d}\left({\tau}_{last}\right)+1\bullet \mathrm{d}\left({\tau}_{last}\right)\ } $$

Each baseline value with its own weight value becomes an input of the sigmoid function to arrange each baseline value as a positive value. A loss function evaluates the squared difference between the amplitude value of the baseline representative point and the output of the sigmoid function. The weight value that demonstrates a smaller gradient value for the loss function is adjusted while iterating the allocated weight values. The adjusted weight values compensate amplitude variations located around the baseline representative point.

### ST-segment deviation evaluation

The ST-segment elevation occurs when the amplitude difference between the J point and the baseline representative point exceeds 0.25 mV for the *V*_2_ and *V*_3_ electrocardiogram channels in males younger than 40 years [36], 0.20 mV for *V*_2_ and *V*_3_ electrocardiogram channels in males older than 40 years, 0.15 mV for *V*_2_ and *V*_3_ electrocardiogram channels in females, and 0.1 mV for other channels [31, 36, 37].

The basic free version of the XML software tool, utilized in the proposed work, which depicts a representative beat from an XML file, offers an amplitude value reading feature between two points of an electrocardiogram. The proposed MI algorithm is tuned by the *V*_2_ channel dataset in an XML file format, which shows that the amplitude difference value between the J point and the baseline representative point is around 0.15 mV. The same *V*_2_ channel dataset in a CSV file format represents the maximum amplitude difference between the J points and baseline representative points as 28, which is equivalent to 0.15 mV. Considering that most amplitude difference values between the J point and baseline representative point is 26 in the CSV file format, the 0.10 mV value in the XML file corresponds to 17 in the CSV file format.

Every dataset in the proposed MI evaluation algorithm follows the experimental subject that shows the amplitude difference value between the J point and the baseline representative point as 0.15 mV in XML and CSV file formats. Then, the amplitude parameter with the value of 17 in the CSV file format corresponds to 0.10 mV.

All other proposed algorithm stages evaluate their own input dataset without additional global parameters, and each algorithm stage is connected by the proposed reinforcement learning architecture. Among the 276 clinical electrocardiograms in CSV format files from 23 patients, electrocardiograms from every channel for the 23 patients were evaluated. Among the 623 clinical electrocardiograms with representative beats from 96 patients, the electrocardiograms exhibited visually certain ST-segment elevation and visually uncertain ST-segment to decide ST-segment elevation are included to be executed by the proposed algorithm. Because clinicians currently determine the condition of MI visually by following the anterior MI criteria, a clinical decision depends on the background knowledge or skill. The visually certain or uncertain electrocardiograms at the channels that ranges from *V*_*1*_ to *V*_*6*_ and the electrocardiograms of their neighbored channels constitute the experimental dataset in as XML file format, considering the purposes of clinical diagnosis. The channel and gender information for each electrocardiogram are considered for the evaluation of ST-segment deviation. When the number of heart beats in the CSV format file that represent the STEMI exceeds 39.9999% of the total number of heart-beats in the same file, the electrocardiogram is considered to be under the condition of MI.

### Impulsive waveform direction evaluation

As the precedent onset of MI tends to inverse waveforms, such clinical electrocardiograms need to be identified. The impulsive waveform direction evaluation stage generates reward values by searching the slope values after the R wave peak; and then the slope values are searched, following the minimum amplitude value after the R wave, with an algorithmic searching range of two times of the average interval between the R wave peak and the minimum amplitude value after the R wave. The slope values are considered as absolute values after the R wave owing to a decreasing trend.

20 $$ {reward}_{Tdir.}=\frac{slope_{\mathit{\max}.}+ avg\left(-1\bullet slope\right)}{2} $$

The next state value of the impulsive waveform direction evaluation stage is evaluated from the approximate entropy. The approximate entropy is obtained by the logarithmic values of the counted numbers, when each datum is smaller than a threshold value for each data subset [38]. The threshold value for the approximate entropy is determined by the absolute maximum between the two sets of slope values at the impulsive waveform direction evaluation stage. When the numbers of the slope values that are below the threshold value are counted, the logarithmic value for the counted number is evaluated. The logarithmic value for the counted number is then divided by the half of the algorithmic searching range at the impulsive waveform direction evaluation stage to evaluate the average value of the logarithmic value for the counted number, equivalent to the next state value of the impulsive waveform direction evaluation stage.

The impulsive waveform direction evaluation stage searches the coefficient *γ* from 0 to 2.5 with an increment number of 0.5. The transition probability is set to 1. The two action values, generated after the R wave and the minimum amplitude value after the R wave, with the same *γ* value become the parameters of the softmax function in Eq. (21) to search the appropriate γ value as shown below.

21 $$ \gamma ={\displaystyle \begin{array}{c} argmin\\ {}\gamma \end{array}}\left[\prod \limits_{i=0}^{i=1} soft\mathrm{m} ax\left({Q}_{\gamma}^i\right)\right] $$

The appropriate *γ* value determines the optimized action value. The minimum value of the result of the multiplied softmax function guarantees that the action value bypasses an abruptly large action value due to the presence of a relatively large slope at the QRS complex or noise component.

The policy becomes the output of the softmax function between the optimized action values of each reward value. The evaluation of the policy follows Eq. (8). The coefficient *α* to be multiplied with the logarithmic function of the policy is searched from 0.5 to 100.5 with an increment number of 1. When the optimized action value appears upon the applied *α* values, the coefficient *α* is selected. Meanwhile, the updated state value is set as an absolute value.

**Fig. 5:**
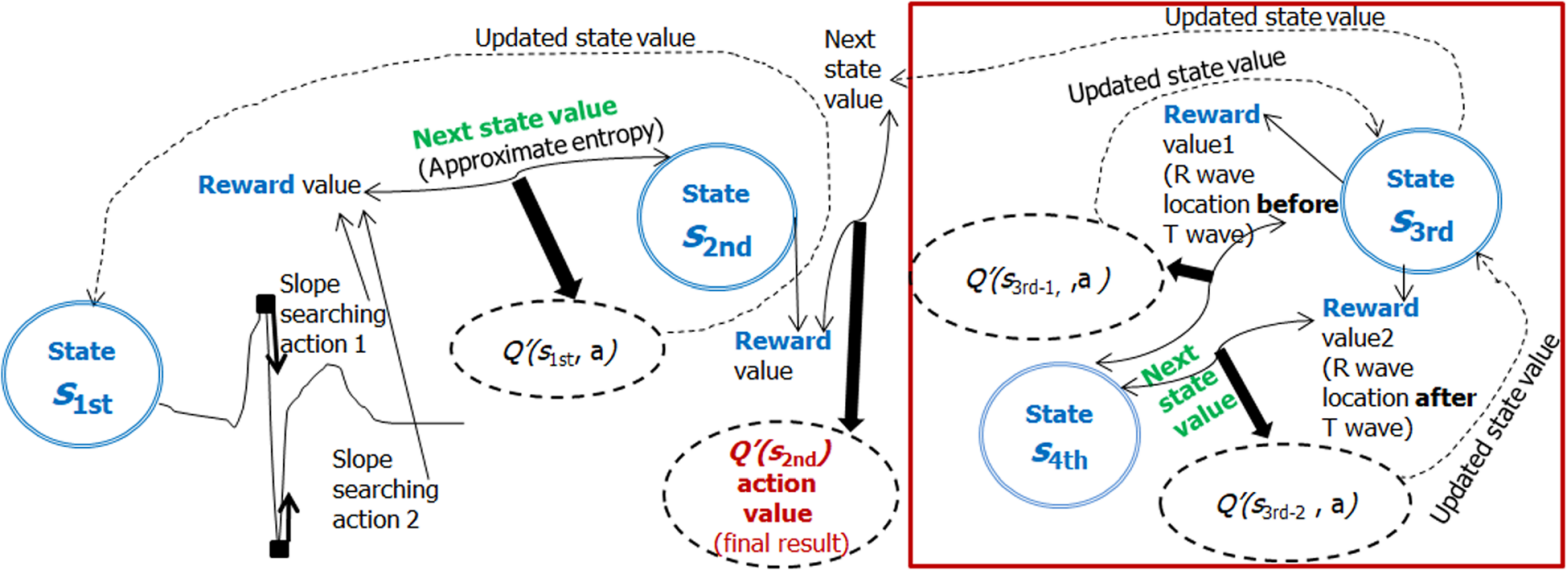
Reinforcement learning environment at impulsive waveform direction evaluation stage

The second state value incorporates the third algorithmic states, as marked in the red square in Fig. 5, which has two reward values. Whenever any larger interval length appears at the prior or posterior position of the T wave, a corresponding reward value becomes the number that is incremented by one. The coefficient *γ* is set to 10 to balance the numerical amount between the reward values and the next state values. The policy applies the softmax function between the two action values, and considers the minimum policy value to be 0.0001 to avoid a deterministic condition such as a probability value of 0. The probabilistic policy optimizes the action values as the optimally updated state values with the coefficient value of *α* as 1.

The reward value of the second algorithmic state in Fig. 5 is added to the next state value of the second algorithmic state, which is equivalent to the updated state value at the incorporated third state, as marked in the red square in Fig. 5. The coefficient *γ* and transition probability are set to 1 to evaluate the final updated state value at the impulsive waveform direction evaluation stage.

The proposed work then intentionally inverses each experimental electrocardiogram, following the amplitude axis, and repeats the QRS complex peak evaluation stage and impulsive waveform direction evaluation stage. The proposed work compares each final updated state value from the non-inversed and inversed electrocardiograms, and selects a higher updated state value to evaluate the direction of the impulsive waveform.

The algorithmic states from the least-first-power approximation stage and impulsive direction waveform stage are interoperable. When the results of the evaluated waveform direction between the least-first-power approximation stage and the impulsive direction waveform stage conflict each other, the ratio between the two stages selects a more numerically deterministic impulsive waveform direction for evaluation, as expressed below.

22 $$ \mathrm{ratio}=\frac{\mid {state\ value}_{non- inversed\ w\mathrm{a} ve}-{state\ value}_{inversed\ wave}\mid }{larger\ state\ value\  at\  numerator}\times 100 $$

The algorithmic state of the ST-segment deviation evaluation stage is also interoperable to the final algorithmic states at the impulsive waveform evaluation stage, because STEMI generates another impulsive wave from the S point to the peak of T wave in an increasing slope. When the ratio of STEMI beats out of the total heart-beats exceeds 20.0%, the impulsive waveform direction is considered to be the non-inversed waveform.

## Results

Our study revealed that the accuracy of the novel hybrid algorithm in interpreting MI was better than that of previous electrocardiograms. When the ratio of heart beats in the CSV file format exceeds 39.9999%, the electrocardiogram is under the condition of MI. The representative beat is obtained in a picture file format from the XML, where the STEMI ratio is either 0 or 1. The MI and the results for impulsive wave shape evaluation from both the CSV files and representative beats are presented in Table 1.

**Table 1:** STEMI and impulsive wave shape evaluation and results

	Input method	Accuracy	Sensitivity	Specificity
STEMI evaluation	CSV file	99.2754%	98.2456%	99.5434%
	Representative beat	99.3579%	99.0099%	99.6875%
Impulsive wave shape evaluation	CSV file	97.4638%	94.5455%	98.1900%
	Representative beat	97.7528%	96.7213%	98.0040%

The interoperability among the least-first-power approximation stage, impulsive wave trend evaluation stage, and ST-segment deviation evaluation stage selects the stage that shows a larger ratio to determine the impulsive wave shape. An additional table file shows the numerical decision of the interoperability between the algorithmic stages in more detail (see Additional file 1).

The MI evaluation results for nine electrocardiograms, among 18 electrocardiograms, from the electrocardiograph at the Yonsei University Gangnam Severance Hospital indicate the same MI evaluation result as that obtained in the proposed work. For the other nine electrocardiograms, the electrocardiograph at the Yonsei University Gangnam Severance Hospital generated wrong results from the evaluation of MI. However, the proposed work discerns that the other nine patients are under MI. The operation time for each CSV format file takes approximately 3.5 min with CPU usage limit around 60% and GPU usage limit around 90% due to an air-cooled thermal solution in a small form factor computer. The operation time for the representative beat data takes approximately 12 s. The proposed work also evaluates MI on the Physionet MIT-BIH ST change database repository [39, 40]. Representative beats are selected as the first heartbeat of each dataset. The accuracy for MI evaluation and impulsive wave shape evaluation was found to be 97.8261% each. Other research works are listed in Table 2.

**Table 2:** Myocardial infarction evaluation results

Model	Accuracy	Sensitivity	Specificity
Hybrid firefly [41]	99.3%	99.97%	98.7%
Hidden Markov [42]	82.50%	85.71%	79.82%
LS-SVM [43]	99.31%	99.62%	98.12%
MLP-NN [44]	96%	–	–
Proposed evaluation	99.36%	99.01%	99.69%

The feature extraction of MI in hybrid firefly [41] is similar to how a firefly in each swarm senses the brightest light, depending on the coefficient values for distant observation conditions. Hybrid firefly [41] utilizes the selected 44 datasets of the Physionet MIT-BIH [40] Physikalisch-Technische Bundesanstalt database. Hidden Markov [42] classifies MI by allocating each algorithmic state that hires a likelihood function at each heartbeat waveform of clinical data from the hospital acknowledged in [42]. The least-squares support-vector machine (LS-SVM) [43] classifier utilizes a radial basis function from wavelet decomposition only at lead *II* that represents a clearer waveform than the other channels. LS-SVM [43] utilized lead *II* datasets that include patients diagnosed with MI and healthy people only, excluding other clinical datasets from the Physionet MIT-BIH [40] Physikalisch-Technische Bundesanstalt database. Multi-layer perceptron neural networks (MLP-NN) [44] utilizes a software program offered by an electrocardiograph for annotated waveforms, a clinical survey, and a genetic algorithm for network training. The experimental results for [44] show that the evaluation accuracy for patients with mention of MI in clinical records is 96%, while that for patients without any mention of MI in the clinical records is 84.5%.

## Discussion

**Fig. 6:**
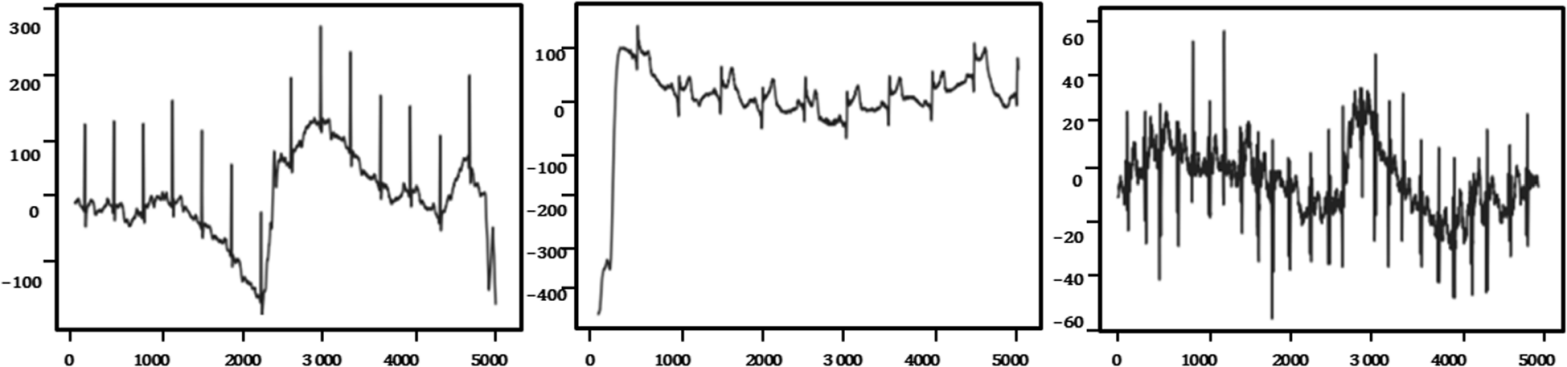
Noise component examples of correctly evaluated STEMI and impulsive wave shape

The experimental dataset in the proposed work includes clinical electrocardiograms. The shape of the impulsive waveform, as a non-inversed or inversed format, depends on the larger amplitude variations between the QRS complex and T wave. The evaluation of impulsive wave shape can be further developed by discerning the QRS complex and T wave separately. Although an electrocardiograph reduces the electrical noise components, including human errors, such as body movements due to muscle contractions, the muscle contractions generate noticeable changes in the electric signal. The proposed work is effective for the evaluation of STEMI and impulsive wave shape under noise components, such as electric signals affected by muscle contractions. Figure 6 represents some examples of electrocardiograms with shivering or muscle rigidity in the experimental dataset.

When the channels at *II*, *III*, and *avF* of an electrocardiogram consecutively represent the STEMI, RV infarction should be considered. RV infarction, as a differential diagnosis, is implemented by reversely located electrocardiograph electrodes. When STEMI occurs at the reversed electrode locations from the original locations at *V*_4_, *V*_5_ and *V*_6_, MI occurs at RV. The proposed method is also effective for reversely located electrocardiograph electrodes. NTG is contraindicated in the setting of an inferior MI with right ventricular involvement because, in this specific situation, the heart is dependent on the preload with blood expansion as a clinical implementation. The reciprocal changes observed at other channels, instead of the channels with MI, implicate the STEMI condition.

### Strength

The novelty of our study is that we developed a novel type of electrocardiogram wave interpretation that enables the detection of acute myocardial infarction (AMI) more accurately by reducing the analytical noise observed in previous electrocardiograms. The development of electrocardiograms is an important issue because it is the first step in detecting AMI before performing any heart specific biomarker test such as cardiac troponins [45]. Electrocardiograms with cardiac troponins has been practice standard for the diagnosis of AMI, early rule-out and risk stratification in patients presenting acute coronary syndrome [46]. There are on-going research works that aim to improve the detection of MI using cardiac troponin assays [47]. However, laboratories often may not be able to report within 2 h. Therefore, improving the diagnostic accuracy of electrocardiograms would be a much faster way to detect AMI as demonstrated in previous studies focused on troponin assays.

Our study is clinically important regarding four reasons, i.e., urgency and high mortality rate of MI, ED circumstances, helping physicians in detecting MI, and prevention of clinically wrong practices. First, we discuss the urgency and high mortality rate of MI. A recent study reported that the five-year mortality rate of MI or myocardial injury is approximately 70%, with a major adverse cardiovascular event rate of 30% [48]. AMI with STEMI is the top priority, which should be urgently referred for thrombolysis or revascularization in real clinical practice. The primary goal in the evaluation of acute chest pain is the prompt identification of AMI. Second, considering the circumstances of the ED, physicians may be falsely reassured if MI is not detected in busy and overcrowded EDs. Physicians might the overlook electrocardiograms in busy circumstances. Crowded EDs should develop systems to quickly identify and prioritize patients with MI. Unrecognized MI is associated with mortality. Thus, risk stratification and early diagnosis is necessary [19]. Third, our result would help physicians in better detecting MI. Fourth, the improved detection of MI would prevent the false administration of NTG to contraindicated patients with inferior MI [45].

### Limitation

The reported high accuracy can cause an over-fitting problem when a programmed algorithmic state keeps searching for data even if the state has already achieved its goal. Most clinical electrocardiograms require all processes in the proposed algorithmic state to be of high accuracy, which is more important than the over-fitting regarding risk and benefit. High accuracy is more important for detecting MI due to the high mortality rate associated with, ED circumstances where physicians could miss the detection of MI, and long waiting time for laboratory results. Our study incorporated the Elliot wave principle, which is used in technical analysis; this principle is a proposed theory in financial fields and can be controversial because it can cause fault localization problems in machine learning. Therefore, further prospective trials are necessary to clarify the clinical roles of the sensitive detection of MI considering the Elliot wave principle.

## Conclusions

Our study revealed that the accuracy of the novel hybrid algorithm, proposed in this algorithm, for interpreting MI was better than that of previous electrocardiogram. Each action value and algorithmic state value offers a numerically tractable reinforcement learning environment. Any algorithmic state can be fused with another algorithmic state if the final algorithmic state value converges explicitly.

The interoperable algorithmic states in reinforcement learning numerically determine the most suitable algorithmic state for clinical electrocardiograms, whereas typical machine learning techniques tend to select the learning algorithms for training and classification, which reflect the characteristics of the selected algorithms.

Although the conceptualized waveforms of electrocardiograms consider the waveforms between the P and QRS complex with a flat line, clinical electrocardiograms incorporates baseline wandering. Baseline wandering can be usually treated by a filter to reduce it; however, the proposed work determines the baseline representative point from a neural network that considers all targeted data between the P and QRS complex. The baseline representative point and the J point practically represent the actual amplitude, which is critical for ST-segment elevation evaluation.

The stopping time determines the impulsive wave trend distribution when an algorithmic decision appears at the impulsive wave trend evaluation stage. The proposed stopping time decision from the downhill U-turn process is widely used for algorithmic states in reinforcement learning such as the impulsive wave trend evaluation stage and baseline approximation stage. Because clinical electrocardiograms incorporates complex oscillations, the proposed downhill U-turn point stage offers versatile applications for complex oscillations at each waveform throughout the P wave, QRS complex and T wave. The proposed downhill U-turn point stage evaluates the cumulative slope variations within a partial waveform. Because the clinical electrocardiograms incorporate complex pathological waveforms, there are frequent chances of convergence to the local maximum or minimum. The cumulative slope variations are robust to locally maximum or minimum slopes, which would cause a fitting problem when processed by a gradient descent or ascent method.

As a clinically important issue, there are some cases of clinical mistakes in the ED, wherein NTG is administered to patients with highly suspected RV infarction. In such cases, the patient’s blood pressure suddenly drops and causes the patient to enter a shock state, which may result in death. Therefore, the proposed work offers assistance in electrocardiogram interpretation that may be helpful in clinical decisions, and prevent inaccurate clinical treatment, such as the administration of contraindicated oral medication to patients with RV infarction. Furthermore, sensitive detection of STEMI may increase the survival rate of patients with AMI.

The results of our hybrid algorithm demonstrate the highest diagnostic accuracy. Furthermore, it can be used as a more accurate screening and diagnostic tool for identifying patients with MI who require urgent treatment.

## Supplementary information

**Additional file 1.** Interoperable algorithm stage selection for impulsive wave shape evaluation. Description of data: Numerical decision of the interoperability among algorithmic stages for impulsive wave shape evaluation.

## Data Availability

Some datasets generated and/or analyzed during the current study are available in the physionet MIT-BIH ST change database repository [32, 33], [10.13026/C2ZW2H]. The other datasets generated and/or analyzed during the current study are not publicly available due to preserved data at Yonsei University Gangnam Severance Hospital, Seoul, Korea, but are available from the corresponding author on a reasonable request.

## Abbreviations

STEMI: ST-Elevation Myocardial Infarction
MI: Myocardial Infarction
NTG: Nitroglycerin
RV: Right Ventricle
ED: Emergency Department
CPU: Central Processing Unit
GPU: Graphics Processing Unit
XML: Extensible Markup Language
CSV: Comma-Separated Values
AMI: Acute Myocardial Infarction

## References

[1] Volna E, Kotyrba M, Janosek M (2017). Pattern recognition and classification in time series data.

[2] Marañon M, Kumral M (2018). Exploring the Elliott wave principle to interpret metal commodity price cycles. Resources Policy.

[3] Dalvi RF, Zago GT, Andreao RV (2016). Heartbeat classification system based on neural networks and dimensionality reduction. Res Biomed Eng.

[4] Dutta S, Chatterjee A, Munshi S (2010). Correlation technique and least square support vector machine combine for frequency domain based ECG beat classification. Med Eng Phys.

[5] Li H, Yuan D, Ma X, Cui D, Cao L (2017). Genetic algorithm for the optimization of features and neural networks in ECG signals classification. Sci Rep.

[6] Alarsan FI, Younes M. Analysis and classification of heart diseases using heartbeat features and machine learning algorithms. J Big Data. 2019;6(81). 10.1186/s40537-019-0244-x.

[7] Brown BD, Badilini F (2005). HL7 aECG Implementation Guide. Regulated Clinical Research Information Management Technical Committee.

[8] Wang S, Xie X, Huang K, Zeng J, Cai Z (2019). Deep reinforcement learning-based traffic signal control using high-resolution event-based data. Entropy..

[9] Marsland S (2009). Machine learning: an algorithmic perspective.

[10] De Saporta B, Dufour F, Nivot C (2017). Partially observed optimal stopping problem for discrete-time Markov processes. 4OR.

[11] Shao K, Tang Z, Zhu Y, Li N, Zhao D (2019). A survey of deep reinforcement learning in video games. arXiv.

[12] Fischer T (2013). On simple representations of stopping times and stopping time sigma-algebras. Stat Probabil Lett.

[13] Tsitsiklis JN, Van Roy B (1999). Optimal stopping of Markov processes: Hilbert space theory, approximation algorithms, and an application to pricing high-dimensional financial derivatives. IEEE Trans Autom Control.

[14] Chevalier E, Ly Vath V, Roch A, Scotti S (2015). Optimal exit strategies for investment projects. J Math Anal Appl.

[15] Detemple J, Tian W, Xiong J (2012). An optimal stopping problem with a reward constraint. Finance Stochastics.

[16] Li L, Linetsky V (2013). Optimal stopping and early exercise: an eigenfunction expansion approach. Oper Res.

[17] Krishnamurthy V, Aprem A, Bhatt S (2018). Multiple stopping time POMDPs: structural results & application in interactive advertising on social media. Automatica..

[18] Edgar GA, Sucheston L (1992). Stopping times and directed processes.

[19] Van der Ende MY, Hartman MHT, Schurer RAJ, van der Werf HW, Lipsic E, Snieder H, van der Harst P (2017). Prevalence of electrocardiographic unrecognized myocardial infarction and its association with mortality. Int J Cardiol.

[20] Kataoka A, Scherrer-Crosbie M, Senior R, Garceau P, Valbuena S, Čelutkienė J, Hastings JL, Cheema AN, Lara A, Srbinovska-Kostovska E, Hessian R, Poggio D, Goldweit R, Saric M, Dajani KA, Kohn JA, Shaw LJ, Reynolds HR, Picard MH (2016). Transient ischemic dilatation during stress echocardiography: an additional marker of significant myocardial ischemia. Echocardiography..

[21] Park JR, Park JE (2017). A T-wave variation characteristics evaluation algorithm for ischemic heart beats. Int J Health Med Sci.

[22] Sánchez C, D’Ambrosio G, Maffessanti F, Caiani EG, Prinzen FW, Krause R, Auricchio A, Potse M (2018). Sensitivity analysis of ventricular activation and electrocardiogram in tailored models of heart-failure patients. Med Biol Eng Comput.

[23] Sutton RS, Barto AG (2018). Reinforcement learning: an introduction.

[24] Watkins CJCH, Sutton RS (1992). Technical note: Q-learning. Reinforcement learning.

[25] Achbany Y, Fouss F, Yen L, Pirotte A, Saerens M (2008). Tuning continual exploration in reinforcement learning: an optimality property of the Boltzmann strategy. Neurocomputing..

[26] Nachum O, Norouzi M, Xu K, Schuurmans D (2017). Bridging the gap between value and policy based reinforcement learning. Proceedings of 31st Conference on Neural Information Processing Systems.

[27] Nachum O, Chow Y, Ghavamzadeh M (2018). Path consistency learning in Tsallis entropy regularized MDPs.

[28] Kalish ML, Griffiths TL, Lewandowsky S (2007). Iterated learning: intergenerational knowledge transmission reveals inductive biases. Psychon Bull Rev.

[29] Sharot T (2017). The influential mind.

[30] Hazinski MF, Samson R, Schexnayder S (2010). 2010 handbook of emergency cardiovascular Care for Healthcare Providers.

[31] Friederich P (2015). ECG monitoring of myocardial ischemia for perioperative care.

[32] Larsen CT, Dahlin J, Blackburn H, Scharling H, Appleyard M, Sigurd B, Schnohr P (2002). Prevalence and prognosis of electrocardiographic left ventricular hypertrophy, ST segment depression and negative T-wave; the Copenhagen City heart study. Eur Heart J.

[33] Rivlin TJ (2003). An introduction to the approximation of functions.

[34] Stauffer HB (2008). Contemporary Bayesian and frequentist statistical research methods for natural resource scientists.

[35] Fry H (2018). Hello world: how to be human in the age of the machine.

[36] Wei EY, Hira RS, Huang HD, Wilson JM, Elayda MA, Sherron SR, Birnbaum Y (2013). Pitfalls in diagnosing ST elevation among patients with acute myocardial infarction. J Electrocardiol.

[37] Coppola G, Carita P, Corrado E, Borrelli A, Rotolo A, Guglielmo M, Nugara C, Ajello L, Santomauro M, Novo S (2013). On behalf of the Italian study Group of Cardiovascular Emergencies of the Italian Society of Cardiology. ST segment elevations: always a marker of acute myocardial infarction?. Indian Heart J.

[38] Delgado-Bonal A, Marshak A (2019). Approximate entropy and sample entropy: a comprehensive tutorial. Entropy..

[39] Albrecht P (1983). S-T segment characterization for long-term automated ECG analysis. MS thesis, Massachusetts institute of technology: Department of Electrical Engineering and Computer Science.

[40] Goldberger AL, Amaral LAN, Glass L, Hausdorff JM, IvanovPCh MRG, Mietus JE, Moody GB, Peng C-K, Stanley HE (2003). PhysioBank, PhysioToolkit, and PhysioNet: components of a new research resource for complex physiologic signals. Circulation..

[41] Kora P (2017). ECG based myocardial infarction detection using hybrid friendly algorithm. Comput Methods Prog Biomed.

[42] Chang P, Lin J, Hsieh J, Weng J (2012). Myocardial infarction classification with multi-lead ECG using hidden Markov models and Gaussian mixture models. Appl Soft Comput.

[43] Kumar M, Pachori RB, Acharya UR (2017). Automated diagnosis of myocardial infarction ECG signals using sample entropy in flexible analytic wavelet transform framework. Entropy..

[44] Kojuri J, Boostani R, Dehghani P, Nowroozipour F, Saki N (2015). Prediction of acute myocardial infarction with artificial neural networks in patients with nondiagnostic electrocardiogram. J Cardiovasc Dis Res.

[45] Furlan L, Rusconi AM, Ceriani E (2017). Five steps for use and interpretation of troponin in the emergency department. Intern Emerg Med.

[46] Apple FS, Sandoval Y, Jaffe AS, Ordonez-Llanos J (2017). Cardiac troponin assays: guide to understanding analytical characteristics and their impact on clinical care. Clin Chem.

[47] McCarthy CP, Raber I, Chapman AR, Sandoval Y, Apple FS, Mills NL, Januzzi JL Jr. Myocardial injury in the era of high-sensitivity cardiac troponin assays: a practical approach for clinicians. Am Med Assoc Cardiol. 2019. 10.1001/jamacardio.2019.2724. 10.1001/jamacardio.2019.2724 31389986

[48] Chapman AR, Shah ASV, Lee KK, Anand A, Francis O, Adamson P, McAllister DA, Strachan FE, Newby DE, Mills NL (2018). Long-term outcomes in patients with type 2 myocardial infarction and myocardial injury. Circulation..

