# Supplementary material for: Myocardial infarction evaluation from stopping time decision toward interoperable algorithmic states in reinforcement learning
Source: BMC Med Inform Decis Mak. 2020 Jun 1;20:99. doi: 10.1186/s12911-020-01133-x (PMC7472590; doi:10.1186/s12911-020-01133-x)
Supplement: Supplementary file 1 — Additional file 1. Interoperable algorithm stage selection for impulsive wave shape evaluation. Description of data: Numerical decision of the interoperability among algorithmic stages for impulsive wave shape evaluation. [file 12911_2020_1133_MOESM1_ESM.docx]

**Table 1. Interoperable algorithm stage selection for impulsive wave shape evaluation in CSV files. (Every case that the results from each algorithmic stage conflict each other is shown.)**

|  |  | **Impulsive waveform (Methods 7)** | |  | **STEMI (Methods 5&6)** | **Least-first-power approximation (Methods 4)** | |  |
| --- | --- | --- | --- | --- | --- | --- | --- | --- |
| **Data#_**  **channel** | **Prevalence algorithm stage# [result]** | **Algorithm result as non-inversed wave(Methods 3)** | **Algorithm result as inversed wave(Methods 3)** | **Amount of ratio of larger value** | **Myocardial infarction evaluation** | **Algorithm result as non-inversed wave** | **Algorithm result as inversed wave** | **Amount of ratio of larger value** |
| 1_V3 | 3[normal] | 10.3671875 | 11.709478 | 11.4633% | 0 | 102420.42 | 67182.43 | 34.4052% |
| 2_3 | 2[normal] | 15.81526 | 25.48804 | 37.9503% | 0.2857143 | 24897 | 24213 | 3.4578% |
| 2_avF | 1[inversed] | 10.639228 | 16.989836 | 37.3800% | 0 | 32560.625 | 20428.125 | 37.2613% |
| 3_v1 | 1[normal] | 29.767673 | 16.551716 | 44.3970% | 0 | 33361.668 | 35524.332 | 5.5561% |
| 3_v4 | 2[normal] | 3.7856596 | 11.435588 | 66.8958% | 0.3334 | 9289.6 | 9254.4 | 0.3789% |
| 4_3 | 1[inversed] | 5.506595 | 25.867546 | 78.7123% | 0 | 18603.6 | 5058.5996 | 72.8158% |
| 4_avR | 1[inversed] | 19.058067 | 20.80054 | 8.3771% | 0 | 16056 | 14820 | 7.6981% |
| 8_v2 | 1[normal] | 48.32267 | 30.515915 | 36.8497% | 0 | 16401.668 | 21278.332 | 22.9185% |
| 9_avL | 3[inversed] | 20.391172 | 13.562566 | 33.4881% | 0 | 8665.8 | 14136.2 | 38.6978% |
| 10_avR | 1[inversed] | 7.4513474 | 22.147583 | 66.3559% | 0 | 388313.2 | 357105.2 | 8.0368% |
| 12_3 | 1[inversed] | 13.793032 | 16.275352 | 15.2520% | 0.2 | 22763.287 | 22214.713 | 2.4099% |
| 12_avR | 1[inversed] | 10.354956 | 29.528912 | 64.9328% | 0 | 33305 | 33207 | 0.2943% |
| 12_v1 | 1[normal] | 12.431835 | 9.897079 | 20.3892% | 0 | 22445.334 | 24866.666 | 9.7373% |
| 13_3 | 3[normal] | 4.5248322 | 15.4595585 | 70.7312% | 0 | 21101.4 | 4095 | 80.5937% |
| 13_v1 | 3[inversed] | 36.059174 | 21.06551 | 11.5083% | 0 | 35395.6 | 46834.797 | 24.4246% |
| 13_v2 | 1[normal] | 48.940296 | 31.90938 | 53.3728% | 0.2 | 14000.285 | 14411.715 | 2.8548% |
| 14_1 | 1[normal] | 24.570753 | 12.805517 | 47.8831% | 0 | 28255.3 | 41894.902 | 32.5567% |
| 14_5 | 1[normal] | 22.828215 | 10.800955 | 52.6859% | 0 | 27451.22 | 31662.78 | 13.3013% |
| 15_1 | 1[normal] | 18.448963 | 4.863075 | 73.6464% | 0 | 29322 | 31978 | 8.3057% |
| 15_3 | 1[inversed] | 10.267803 | 15.476635 | 33.6561% | 0 | 139909.88 | 102935.875 | 26.4270% |
| 15_6 | 3[normal] | 10.11025 | 13.975938 | 27.6596% | 0 | 93631.125 | 56083.125 | 40.1021% |
| 15_7 | 2[normal] | 38.151356 | 11.266462 | 70.4690% | 0.3333 | 28106.375 | 28363.625 | 9.9070% |
| 16_avF | 3[inversed] | 19.975159 | 18.698338 | 6.3920% | 0 | 10740.834 | 11569.166 | 7.1598% |
| 18_avR | 1[inversed] | 28.59982 | 38.50256 | 25.7197% | 0.125 | 38606.797 | 38405.203 | 0.5222% |
| 18_avF | 1[inversed_WrongResult!]=>normal! | 31.976635 | 39.66866 | 19.3907% | 0 | 36131.82 | 34248.148 | 5.2132% |
| 19_3 | 3[normal] | 42.941948 | 43.52636 | 1.3427% | 0 | 41708.098 | 37893.9 | 9.1450% |
| 20_avL | 3[normal] | 10.399239 | 13.409419 | 22.4483% | 0 | 20783.713 | 15416.857 | 25.8224% |
| 20_avF | 1[normal] | 21.44948 | 13.632286 | 36.4447% | 0 | 30356.555 | 33165.445 | 8.4693% |
| 20_v5 | 1[normal] | 18.593414 | 11.315913 | 39.1402% | 0 | 6055.4995 | 7282.8335 | 16.8524% |
| 20_v6 | 1[normal] | 15.618597 | 9.761462 | 37.5010% | 0 | 20614.375 | 28775.875 | 28.3623% |
| 21_2 | 3[normal] | 18.123432 | 21.18237 | 14.4410% | 0 | 14058.666 | 10725.334 | 23.7102% |
| 21_3 | 1[inversed] | 19.04417 | 28.816896 | 33.9132% | 0 | 18438.332 | 16991.668 | 7.8460% |
| 21_avF | 2[STEMI] | 14.48586 | 20.778065 | 30.2829% | 0.4 | 80318.85 | 36034.85 | 55.1353% |
| 21_v5 | 1[inversed] | 51.700302 | 129.84773 | 60.1839% | 0 | 13627.5 | 9643.5 | 29.2350% |
| 22_3 | 1[inversed] | 13.968173 | 16.470028 | 15.1904% | 0 | 18554 | 17294 | 6.7909% |
| 22_avR | 3[normal] | 12.6592865 | 24.326149 | 47.9602% | 0 | 63257.625 | 21208.875 | 66.4722% |
| 22_avF | 1[normal] | 18.29 | 12.898081 | 29.4801% | 0 | 24975.143 | 17728.572 | 29.0151% |
| 23_avR | 1[inversed] | 6.649335 | 24.816288 | 73.2058% | 0 | 25756.43 | 20733.572 | 19.5014% |
| 23_v1 | 2[STEMI] | 21.54606 | 33.106533 | 34.9190 | 0.5 | 37572.668 | 11636 | 69.0307% |
| 23_v3 | 2[STEMI] | 7.1819077 | 29.57167 | 75.7136% | 0.8333 | 72935.875 | 21857.625 | 70.0317% |

**Table 2. Interoperable algorithm stage selection for impulsive wave shape evaluation in representative beat. (Every case that the results from each algorithmic stage conflict each other is shown.)**

|  |  | **Impulsive waveform** | |  | **STEMI** | **Least-first-power approximation** | |  |
| --- | --- | --- | --- | --- | --- | --- | --- | --- |
| **Data#_**  **channel** | **Prevalence algorithm stage#**  **[result]** | **Algorithm result as non-inversed wave** | **Algorithm result as inversed wave** | **Amount of ratio of larger value** | **Myocardial infarction evaluation** | **Algorithm result as non-inversed wave** | **Algorithm result as inversed wave** | **Amount of ratio of larger value** |
| 12_v2 | 3[inversed] | 25.372334 | 20.629398 | 18.69% | 0 | 33 | 55 | 40.0% |
| 32_2 | 1[normal] | 8.13405 | 1.6418407 | 79.82% | 0 | 78 | 92 | 15.22% |
| 36_avR | 1[inversed] | 6.959538 | 13.784922 | 49.51% | 0 | 38 | 30 | 21.05% |
| 39_v6 | 1[normal] | 61.610775 | 26.444105 | 54.03% | 0 | 72 | 78 | 7.69% |
| 46_v4 | 3[inversed] | 45.627155 | 39.868202 | 12.62% | 0 | 2 | 44 | 95.45% |
| 47_v1 | 3[normal] | 5.280442 | 8.595556 | 38.57% | 0 | 88 | 4 | 95.45% |
| 48_v1 | 1[inversed] | 4.4926186 | 10.865887 | 58.65% | 0 | 69 | 33 | 52.17% |
| 48_v2 | 3[normal] | 8.829383 | 11.019634 | 19.88% | 0 | 71 | 9 | 87.32% |
| 49_v3 | 3[inversed] | 35.591034 | 32.909504 | 69.04% | 0 | 10 | 42 | 76.19% |
| 50_v1 | 1[inversed] | 6.3513875 | 13.339608 | 52.39% | 0 | 442 | 320 | 27.60% |
| 52_v1 | 3[inversed_WrongResult!]=>STEMI! | 27.882282 | 17.60785 | 36.85% | 0 | 112 | 204 | 45.10% |
| 53_v1 | 3[normal] | 8.924817 | 10.899347 | 18.12% | 0 | 59 | 15 | 74.58% |
| 55_v3 | 3[normal] | 18.098568 | 18.268427 | 0.93% | 0 | 74 | 44 | 40.54% |
| 57_v2 | 3[inversed] | 22.447004 | 21.067547 | 6.15% | 0 | 5 | 73 | 93.15% |
| 57_v3 | 3[inversed] | 29.37666 | 22.481237 | 23.47% | 0 | 18 | 36 | 50.0% |
| 59_2 | 1[inversed_WrongResult!]=>normal! | 20.119343 | 50.63066 | 60.26% | 0 | 100 | 62 | 38.0% |
| 59_avF | 3[normal] | 20.599604 | 21.230677 | 2.97% | 0 | 107 | 71 | 33.64% |
| 61_v2 | 3[normal] | 21.572166 | 21.585926 | 0.06% | 0 | 30 | 2 | 93.33% |
| 63_v4 | 1[normal] | 47.397537 | 18.483612 | 61.0% | 0 | 23 | 43 | 46.51% |
| 65_v3 | 3[normal] | 18.692507 | 23.45565 | 20.31% | 0 | 52 | 8 | 84.62% |
| 67_avL | 3[normal] | 3.450901 | 4.3102803 | 19.94% | 0 | 95 | 69 | 27.37% |
| 67_v2 | 3[normal] | 17.293673 | 17.846754 | 3.10% | 0 | 79 | 59 | 25.32% |
| 67_v3 | 3[normal] | 14.606373 | 17.608042 | 17.05% | 0 | 68 | 32 | 52.94% |
| 69_v4 | 3[inversed] | 27.247034 | 26.494326 | 2.76% | 0 | 42 | 66 | 36.36% |
| 71_v6 | 3[normal] | 2.4477851 | 2.558783 | 4.34% | 0 | 1278 | 1216 | 4.85% |
| 74_v2 | 3[inversed] | 18.63342 | 18.5686 | 0.35% | 0 | 50 | 68 | 27.47% |
| 74_v4 | 3[inversed] | 20.477545 | 19.386145 | 5.33% | 0 | 30 | 80 | 62.50% |
| 74_v5 | 3[inversed] | 35.5912 | 11.548839 | 67.55% | 0 | 13 | 131 | 90.08% |
| 74_v6 | 1[normal] | 74.22374 | 12.106213 | 83.69% | 0 | 78 | 126 | 38.10% |
| 78_v1 | 3[normal_WrongResult!]=>inversed! | 10.9776125 | 11.432689 | 3.81% | 0 | 164 | 150 | 8.54% |
| 85_v1 | 3[normal] | 14.657491 | 16.714073 | 12.30% | 0 | 92 | 68 | 26.09% |
| 86_v3 | 3[inversed_WrongResult!]=>normal! | 34.046673 | 26.686493 | 21.62% | 0 | 8 | 34 | 76.47% |
| 89_v2 | 1[inversed] | 8.824764 | 51.81315 | 82.97% | 0 | 119 | 81 | 31.93% |
| 98_v3 | 1[inversed] | 13.478481 | 18.921804 | 28.77% | 0 | 80 | 62 | 22.50% |
| 100_v1 | 1[inversed] | 3.3981512 | 6.1746306 | 44.97% | 0 | 54 | 50 | 7.41% |
| 100_v3 | 1[inversed] | 2.7687387 | 10.7333355 | 74.20% | 0 | 131 | 115 | 12.21% |
| 100_v4 | 3[normal_WrongResult!]=>inversed! | 2.154186 | 7.7397795 | 72.17% | 0 | 292 | 24 | 91.78% |
| 109_v5 | 1[inversed_WrongResult!]=>normal! | 15.394092 | 19.29134 | 20.20% | 0 | 80 | 66 | 17.50% |
| 111_v4 | 1[normal] | 13.889108 | 11.559775 | 16.77% | 0 | 221 | 243 | 9.05% |
| 114_avL | 1[normal] | 15.30433 | 12.062936 | 21.18% | 0 | 99 | 101 | 1.98% |
| 114_v2 | 1[inversed] | 19.356928 | 22.592852 | 14.32% | 0 | 258 | 24 | 14.29% |
| 114_v3 | 3[inversed] | 19.968262 | 17.954674 | 10.08% | 0 | 16 | 28 | 42.86% |
| 114_v4 | 1[inversed] | 16.05428 | 23.07625 | 30.43% | 0 | 17 | 21 | 19.05% |
| 115_v2 | 1[normal] | 7.840416 | 2.297382 | 70.70% | 0 | 63 | 69 | 8.70% |
| 115_v6 | 3[inversed] | 12.500918 | 8.072758 | 35.42% | 0 | 43 | 217 | 80.18% |
| 118_v1 | 1[inversed] | 7.198202 | 15.676199 | 54.08% | 0 | 273 | 143 | 47.62% |
| 120_v5 | 1[inversed] | 6.3671265 | 38.76365 | 83.57% | 0 | 167 | 65 | 61.08% |
| 120_v6 | 3[normal] | 6.765864 | 23.16766 | 70.80% | 0 | 142 | 16 | 88.73% |
| 120_2 | 3[normal] | 8.478862 | 21.991499 | 61.44% | 0 | 162 | 2 | 98.77% |
| 120_3 | 3[normal] | 7.149002 | 10.852772 | 34.13% | 0 | 139 | 49 | 64.75% |
| 120_avF | 3[normal] | 7.76494 | 22.750126 | 65.87% | 0 | 164 | 20 | 87.80% |
| 121_v2 | 1[inversed] | 3.907843 | 5.2765617 | 25.94% | 0 | 5563 | 5481 | 1.47% |
| 121_3 | 3[normal] | 5.897361 | 7.4413757 | 1.54% | 0 | 5391 | 5207 | 3.41% |
| 122_3 | 3[inversed] | 12.74974 | 12.333604 | 3.26% | 0 | 66 | 76 | 13.16% |
| 122_v2 | 1[inversed_WrongResult!]=>normal! | 4.684796 | 12.5590515 | 62.70% | 0 | 49 | 31 | 36.79% |
| 123_avR | 1[inversed] | 10.130782 | 14.024913 | 27.77% | 0 | 126 | 116 | 7.94% |
| 123_avL | 1[inversed] | 4.097298 | 9.929306 | 58.74% | 0 | 92 | 50 | 45.65% |
| 125_avF | 1[inversed] | 1.2655008 | 1.7612619 | 28.15% | 0 | 157 | 117 | 25.48% |
| 134_v1 | 1[inversed] | 6.474638 | 10.799969 | 40.05% | 0 | 53 | 44 | 37.34% |
| 137_2 | 1[normal] | 9.908236 | 6.3157196 | 36.26% | 0 | 88 | 118 | 25.42% |
| 138_1 | 3[normal] | 6.01918 | 9.780535 | 38.46% | 0 | 94 | 16 | 82.98% |
| 138_avL | 3[normal] | 5.148961 | 6.6733146 | 22.84% | 0 | 64 | 12 | 81.25% |
| 139_v2 | 1[normal] | 18.809937 | 6.676965 | 64.50% | 0 | 86 | 60 | 30.23% |
| 139_v4 | 1[normal] | 20.454325 | 1.6278368 | 92.04% | 0 | 53 | 179 | 70.39% |
| 140_v1 | 3[normal] | 8.362994 | 12.225776 | 31.60% | 0 | 43 | 27 | 37.21% |
| 141_v2 | 3[normal] | 5.1054807 | 17.816242 | 71.34% | 0 | 65 | 7 | 89.23% |
| 142_v2 | 3[normal] | 10.164193 | 17.409725 | 41.62% | 0 | 25 | 11 | 56.0% |
| 144_v5 | 1[normal] | 42.69577 | 27.440302 | 35.73% | 0 | 34 | 46 | 26.09% |
| 146_v5 | 3[normal] | 11.166139 | 14.420975 | 22.57% | 0 | 175 | 14 | 92.57% |
| 147_v2 | 3[normal] | 22.143166 | 22.354517 | 0.95% | 0 | 72 | 24 | 66.67% |
| 148_v1 | 1[inversed_WrongResult!]=>normal! | 11.258341 | 15.732902 | 28.44% | 0 | 50 | 38 | 24% |
| 151_2 | 3[normal] | 1.9907498 | 4.190239 | 52.49% | 0 | 69 | 21 | 69.57% |
| 151_v6 | 1[inversed] | 7.31157 | 15.675085 | 53.36% | 0 | 20 | 14 | 30.0% |
| 153_v2 | 1[inversed] | 8.5365305 | 33.67112 | 67.75% | 0 | 62 | 26 | 58.06% |
